# Calycosin Safeguards Mitochondrial Genomic Stability and Iron Homeostasis via the NRF2/NRF1-TFAM Axis to Mitigate Doxorubicin-Induced Cardiotoxicity

**DOI:** 10.3390/antiox15091058

**Published:** 2026-08-25

**Authors:** Huan Chen, Xichen Li, Yishuang Yuan, Shuyi Wang, Changxu Xie, Ran Tian, Qianbin Sun, Tianjiao Shi, Xiaomin Yang, Dongqing Guo, Yong Wang, Qiyan Wang

**Affiliations:** 1 School of Life Sciences, Beijing University of Chinese Medicine, Beijing 100029, China; 2 College of Chinese Medicine, Beijing University of Chinese Medicine, Beijing 100029, China; 3 Key Laboratory of TCM Syndrome and Formula (Beijing University of Chinese Medicine), Ministry of Education, Beijing 100029, China; 4 Beijing Key Laboratory of TCM Syndrome and Formula, Beijing University of Chinese Medicine, Beijing 100029, China

**Keywords:** doxorubicin-induced cardiotoxicity, calycosin, mitochondrial dysfunction, oxidative stress, NRF2

## Abstract

Doxorubicin (DOX) remains a cornerstone of chemotherapy but is limited by dose-dependent doxorubicin-induced cardiotoxicity (DIC). Recent evidence suggests that mitochondrial dysfunction, particularly oxidative stress and ferroptosis, drives DIC progression. While the flavonoid Calycosin (CAL) is known for its cardioprotective properties, the precise mechanism by which it maintains mitochondrial homeostasis remains elusive. This study aimed to investigate the effects of CAL against DIC and to explore the mechanisms by which CAL regulates mitochondrial integrity and iron homeostasis. Using both in vitro H9c2 cardiomyocytes and in vivo C57BL/6 mice, we demonstrate that CAL significantly restores cardiac contractility and reduces myocardial damage. Transcriptomic analysis indicated that CAL protected mitochondrial function and inhibited ferroptosis. Transmission Electron Microscopy (TEM) showed that CAL preserved mitochondrial structure. Assessments of the lipid peroxidation marker malondialdehyde (MDA) and superoxide dismutase (SOD) confirmed that CAL alleviated oxidative stress, while 8-OHdG staining verified its restoration of mitochondrial DNA integrity. Molecular docking identified the affinity of CAL with NRF2 and NRF1. CAL activated the NRF2/NRF1-TFAM axis both in vivo and in vitro, promoting mitochondrial biogenesis and respiratory function, while regulating iron homeostasis via FTMT and FPN. Knockdown or inhibition of NRF2/NRF1 abolished these protective effects of CAL. Our findings suggest that CAL acts as a dual-regulator of mitochondrial transcriptional machinery and iron metabolism, offering a novel therapeutic strategy to safeguard mitochondria against ferroptosis.

## 1. Introduction

Doxorubicin (DOX), a potent anthracycline chemotherapeutic agent, demonstrates remarkable antitumor efficacy; however, its clinical use is limited due to dose-dependent and cumulative cardiotoxic effects [1]. The development of heart failure remains a major cause of morbidity among cancer survivors treated with DOX [2]. Dexrazoxane, the only FDA-approved cardioprotectant [3,4], is limited by safety concerns [5,6], highlighting the pressing need to develop safer therapeutic approaches.

The development of DOX-induced cardiotoxicity (DIC) involves multiple pathological mechanisms, among which mitochondrial impairment and dysregulated iron metabolism are considered key contributors to cardiomyocyte injury [7,8,9]. DOX directly impairs the mitochondrial electron transport chain (ETC), damages mitochondrial DNA (mtDNA), and induces overwhelming oxidative stress [10,11,12]. Concurrently, DOX disrupts cellular iron homeostasis, causing an accumulation of labile iron that accelerates lipid peroxidation and promotes ferroptotic cell death [13,14,15]. These processes engage in a vicious cycle: mitochondrial damage promotes iron-mediated toxicity, while iron overload exacerbates mitochondrial injury. However, the upstream regulatory mechanisms capable of coordinately mitigating both mitochondrial collapse and ferroptosis remain poorly defined, representing a potential therapeutic opportunity.

Calycosin (CAL), a major bioactive isoflavone from *Astragalus membranaceus*, has demonstrated antioxidant and potential cardioprotective properties [16,17,18], yet its precise molecular mechanism against DIC is unclear. To identify the targets of CAL, we performed transcriptomic profiling in a murine DIC model. This analysis revealed that CAL treatment specifically reversed DOX-induced dysregulation of genes involved in mitochondrial energy metabolism and the ferroptosis pathway. This finding suggested that CAL might act on a common upstream regulator coordinating these two pathological modules.

We focused on the potential NRF2/NRF1 transcriptional axis. NRF2 is a well-established master regulator of cellular antioxidant responses and a key suppressor of ferroptosis [19,20,21]. In comparison, NRF1 functions as a key transcription factor responsible for regulating nuclear-encoded mitochondrial genes and mitochondrial biogenesis [22,23]. Additionally, mitochondrial transcription factor A (TFAM), a critical downstream effector of NRF1, is indispensable for mtDNA transcription, replication, and maintenance of mitochondrial genome stability [24,25]. Thus, the NRF2/NRF1-TFAM axis represents a plausible, integrated signaling hub capable of simultaneously enhancing cellular antioxidant/iron-handling capacity (via NRF2) and restoring mitochondrial genomic integrity and bioenergetic function (via NRF1/TFAM). Supporting this hypothesis, in silico molecular docking predicted high-affinity binding of CAL to both NRF2 and NRF1, suggesting its potential as a direct modulator of this axis.

We therefore proposed that CAL protects against DIC by activating the NRF2/NRF1-TFAM signaling cascade, thereby orchestrating a dual defense that concurrently preserves mitochondrial function and inhibits ferroptosis. We show that this axis is essential for CAL-mediated cardioprotection through extensive in vitro and in vivo functional tests. Our work elucidates a novel, coordinated mechanism by which a bioactive compound derived from traditional Chinese medicine mitigates DIC, revealing the therapeutic potential of simultaneously targeting mitochondrial integrity and ferroptosis via the integrated NRF2/NRF1-TFAM pathway—a strategy that may overcome the limitations of concurrent approaches.

## 2. Materials and Methods

### 2.1. Drugs

Calycosin was obtained from Chengdu Desite Biotech Co., Ltd. (Chengdu, China), while DOX and ML385 were acquired from Shanghai Yuan Ye Biotechnology Co., Ltd. (Shanghai, China). Dexrazoxane was supplied by GLPBIO (Montclair, CA, USA).

### 2.2. Animal Treatment

Two separate cohorts of mice were used in this study, totaling 74 animals. The first cohort consisted of 50 male C57BL/6 mice (6–8 weeks old, 20 ± 2 g) from Beijing SPF Biotechnology Co., Ltd. (Beijing, China), which, after 3 days of acclimatization, were randomly assigned to five groups (*n* = 10): control, DOX, DOX + low-dose calycosin, DOX + high-dose calycosin, and DOX + dexrazoxane groups. International ethical rules were followed when conducting animal investigations. DIC induction was performed as per established methods [19]. In brief, DOX was administered intravenously via tail vein injection at a dose of 6 mg/kg on days 6 and 9. According to previous reports [23], calycosin was administered orally at a low (40 mg/kg/day) and high (80 mg/kg/day) doses throughout the entire experimental period. The mice received calycosin pretreatment for 5 days before the first DOX injection. DOX was administered intravenously to the model group via the tail vein, while the control group received an equivalent volume of saline (0.9% NaCl). Dexrazoxane (40 mg/kg) was administered via tail vein injection 30 min before each DOX administration on days 6 and 9, resulting in a cumulative dose of 80 mg/kg. At the experimental endpoint, survival rates were 100% (10/10), 60% (6/10), 90% (9/10), 100% (10/10), and 90% (9/10) in the control, DOX, DOX + low-dose calycosin, DOX + high-dose calycosin, and DOX + dexrazoxane groups, respectively. Six surviving mice per group were randomly selected for most endpoint analyses. Investigators responsible for outcome assessment and data analysis were blinded to group allocation.

To investigate whether calycosin protects the heart via NRF2, 24 additional mice were randomly divided into four groups (*n* = 6): control, DOX, DOX + calycosin, and DOX + ML385 (NRF2 inhibitor). Calycosin was administered via gavage throughout the entire process (80 mg/kg/day). The NRF2 inhibitor ML385 was administered intraperitoneally 30 min before calycosin every other day.

### 2.3. Echocardiography Assessment

Mice were subjected to mild anesthetic (1–2% isoflurane) and dynamic echocardiographic images were obtained and recorded for 30 s at the short-axis muscle level using the Vevo 2100 (FUJIFILM VisualSonics Inc., Toronto, ON, Canada) ultrasound system. Analysis of ejection fraction (EF) and fractional short-axis strain (FS), was performed across three consecutive cardiac cycles to obtain echocardiographic data.

### 2.4. CK-MB, LDH Levels Assessment

After cardiac function assessment, ocular blood was collected, the animals were euthanized, and samples were stored at 4 °C. Serum was prepared and kept at 4 °C, and CK-MB and LDH levels were determined following the kit manual (Solarbio, Beijing, China).

### 2.5. Histopathologic Assay

After collecting ocular blood from mice, the heart of the mouse was removed and rinsed off any excess blood with physiological saline. Following fixation with 4% paraformaldehyde, the heart tissue samples were paraffin-embedded. Sections were subsequently stained with hematoxylin and eosin (H&E). The sections were observed under an optical microscope at a magnified at 200×, and the images were recorded.

### 2.6. Transmission Electron Microscopy

The differences in cardiac mitochondria across groups were observed using TEM imaging. Briefly, small myocardial tissue specimens (1 mm^3^) were fixed in 4% paraformaldehyde at 4 °C for 2 h, post-fixed in 1% osmium tetroxide for 1 h, dehydrated through a graded ethanol series, embedded, and sectioned ultrathin (60–80 nm). Lead citrate and an aqueous solution saturated with 2% uranyl acetate were used for staining for 15 min each, TEM was used to analyze the myocardial ultrastructure. Images were acquired and analyzed.

### 2.7. Measurement of SOD, MDA Levels

Cardiac tissues were homogenized in extraction buffer and centrifuged at 8000× *g* for 10 min at 4 °C. The protein concentrations of the resulting supernatants were determined using a bicinchoninic acid (BCA) protein assay. SOD activity was measured using a WST-1 (Water-Soluble Tetrazolium 1)-based assay kit (BC5165, Solarbio, Beijing, China). The reaction mixtures were incubated at 37 °C for 30 min, and absorbance was measured at 450 nm. SOD activity was calculated from the inhibition rate according to the manufacturer’s instructions and expressed as U/mg protein. MDA content was determined using a thiobarbituric acid-based assay kit (BC0020, Solarbio, Beijing, China). The reaction mixtures were heated at 100 °C for 60 min, cooled on ice, and centrifuged at 10,000× *g* for 10 min at room temperature. Absorbance was measured at 532 and 600 nm, and MDA content was calculated using the blank-corrected difference between the two absorbance values and expressed as nmol/mg protein.

### 2.8. Immunofluorescence

Immunofluorescence staining was performed using an anti-NRF2 antibody (A21508, ABclonal, Wuhan, China), an anti-8-OHdG antibody (sc-393871, Santa Cruz Biotechnology, Dallas, TX, USA) and an anti-Tom20 antibody (A19403, ABclonal, Wuhan, China) on tissue sections. Fluorescent signals were captured with a fluorescence microscope.

### 2.9. Transcriptomic Analysis

Heart tissues stored in RNA were later sent to OE Biotech (Shanghai, China) for sequencing. Total RNA was extracted with TRIzol following the manufacturer’s protocol. RNA purity and concentration were measured by a NanoDrop 2000 (Thermo Scientific, Wilmington, DE, USA), and integrity was evaluated using an Agilent 2100 Bioanalyzer (Agilent Technologies, Santa Clara, CA, USA). The TruSeq Stranded mRNA LT Sample Prep Kit (Illumina, San Diego, CA, USA) was used to create a sequencing library in accordance with the manufacturer’s instructions. An Illumina HiSeq X Ten platform was used for sequencing, producing 150 bp paired-end reads. Differentially expressed genes (DEGs) were defined as genes with expression variations of ≥2-fold and a *p*-value < 0.05. Z-score normalization was employed to visualize DEGs in a heatmap. Enrichment analysis of DEGs was performed using Gene Ontology (GO) and the Kyoto Encyclopedia of Genes and Genomes (KEGG) via R (version 3.2.0), based on the hypergeometric distribution.

### 2.10. Molecular Docking and Dynamics Simulation

Molecular docking and molecular dynamics (MD) simulations were performed to investigate the interaction between calycosin and target proteins. The crystal structures of NRF2 and NRF1 were obtained from the Protein Data Bank, while the two-dimensional structure of calycosin was retrieved from PubChem and converted into a three-dimensional conformation using ChemOffice. All structures were prepared using AutoDock Tools 1.5.7, including hydrogen addition and removal of water molecules, followed by conversion into PDBQT format. Docking was carried out using AutoDock Vina 1.2.7, and binding affinities were evaluated based on calculated binding energies. The optimal binding conformations were visualized using PyMOL 2.6.2.

MD simulations were conducted with GROMACS 2022.2. The Amber14SB force field was applied to proteins, while ligand parameters were generated using the GAFF force field with RESP charges calculated via Multiwfn and ORCA. The protein–ligand complex was solvated in a dodecahedral box with TIP3P water and neutralized with counter ions. After energy minimization, the system underwent equilibration under NVT and NPT conditions, followed by a 100 ns production run. Trajectories were analyzed using PyMOL and VMD.

### 2.11. Cell Viability Assay

To establish an in vitro cardiotoxicity model, H9c2 (Beijing EallBio Biomedical Technology Co., Ltd., Beijing, China) or 4T1 (Shanghai Fuheng Biotechnology Co., Ltd., Shanghai, China) cells were exposed to 1 μmol/L DOX for 24 h. After treatment, the culture medium was removed, and 10% CCK-8 solution was added to each well followed by incubation at 37 °C for 2 h. Absorbance was then measured at 450 nm according to the manufacturer’s instructions (Dalian Meilun Biotechnology Co., Ltd., Dalian, China).

### 2.12. Transfection of NRF2 and NRF1

The transfection process was carried out in a cell culture incubator for a total of 48 h. After a 24-h transfection period, calycosin (16 μmol/L) was added. Subsequently, the medium was removed and replaced with fresh medium containing calycosin (16 μmol/L) and DOX (1 μmol/L). The siRNA sequences (5′−3′) were designed as follows: siNRF2: 5′-GCAAGAAGCCAGATACAAAGA-3′; siNRF1: 5′-GGCTGCTGGGAAGTACAAACA-3′. The siRNAs used in H9c2 cells were specifically designed to target the Rattus norvegicus sequences of NRF2 and NRF1.

### 2.13. Measurement of Fe^2+^ Levels

The cellular Fe^2+^ levels were evaluated using the FerroOrange fluorescent probe [16]. Specifically, cells were supplemented with a 1 μmol/L working solution of FerroOrange (Dojindo Laboratories, Kumamoto, Japan) in serum-free media and treated at 37 °C for 30 min. Fluorescence images were subsequently captured using a fluorescence microscope.

### 2.14. Mitochondrial ROS Evaluation

The H9c2 cells to be tested were seeded in confocal dishes and subjected to experiments when reaching 70–80% confluency. According to the kit instructions, prepared working solutions were added, followed by staining with Mito-Tracker Green (Beyotime Biotechnology, Shanghai, China) and Mito-SOX Red reagents (MedChemExpress LLC, Monmouth Junction, NJ, USA) for 30 min each. After staining, the solutions were removed and replaced with fresh prewarmed culture medium (37 °C). Images were acquired using a laser scanning confocal microscope.

### 2.15. Detection of JC-1

H9c2 cells were cultured to the desired density, pretreated with calycosin (16 μmol/L) for 24 h, followed by DOX exposure for an additional 24 h. After medication administration, cells were incubated with JC-1 staining solution at 37 °C in the dark for 30 min to assess mitochondrial membrane potential. Fluorescence images were obtained using a laser confocal microscope.

### 2.16. Western Blot Assay

Tissue or H9c2 cells were placed in PIPA lysis buffer solution and lysed for 30 min. After that, the homogenized cardiac tissue was put into a centrifuge tube that had been chilled beforehand, and it was centrifuged for ten minutes at 12,000× *g*. Total protein in the supernatant was collected and quantified, followed by separation via SDS-PAGE and transfer onto PVDF membranes for immunoblotting according to standard procedures. Protein bands were detected by chemiluminescence, and densitometric analysis was performed using Image Lab software (version 6.1.0 Build 7, Bio-Rad Laboratories, Inc., Hercules, CA, USA). The intensity of each target protein band was normalized to the corresponding β-actin signal, and the normalized values were used for statistical analysis. The following primary antibodies were employed for immunoblotting analysis: anti-NRF2 (A21508, ABclonal, Wuhan, China), anti-GPX4 (CQA1094, Cohesion, Suzhou, China), anti-FTMT (CQA6550, Cohesion, Suzhou, China), anti-NRF1 (66832-1-Ig, Proteintech, Rosemont, IL, USA), anti-TFAM (82745-1-RR, Proteintech, Rosemont, IL, USA), anti-NDUFB8 (A19732, ABclonal, Wuhan, China), anti-COX1 (A23123, ABclonal, Wuhan, China), anti-SDHB (A23832, ABclonal, Wuhan, China), anti-ATP5A1 (A11217, ABclonal, Wuhan, China), anti-β-actin (66009-1-Ig, Proteintech, Rosemont, IL, USA) as loading control, and anti-FPN (NBP1-21502SS, NOVUS, Centennial, CO, USA). All antibodies were diluted according to manufacturers’ recommended protocols for optimal detection sensitivity.

### 2.17. Statistical Analysis

GraphPad Prism 9.0 was used for statistical analysis. Data are presented as mean ± standard deviation (SD). Before parametric analysis, data normality was assessed using the Shapiro–Wilk test, and homogeneity of variance was evaluated using the Brown–Forsythe test. Data satisfying these assumptions were analyzed using one-way analysis of variance (ANOVA), followed by Tukey’s multiple-comparisons post hoc test. A value of *p* < 0.05 was considered statistically significant. All experiments were performed with at least three independent replicates.

## 3. Results

### 3.1. Calycosin Attenuates DOX-Induced Myocardial Damage and Cardiac Dysfunction

**Figure 1:**
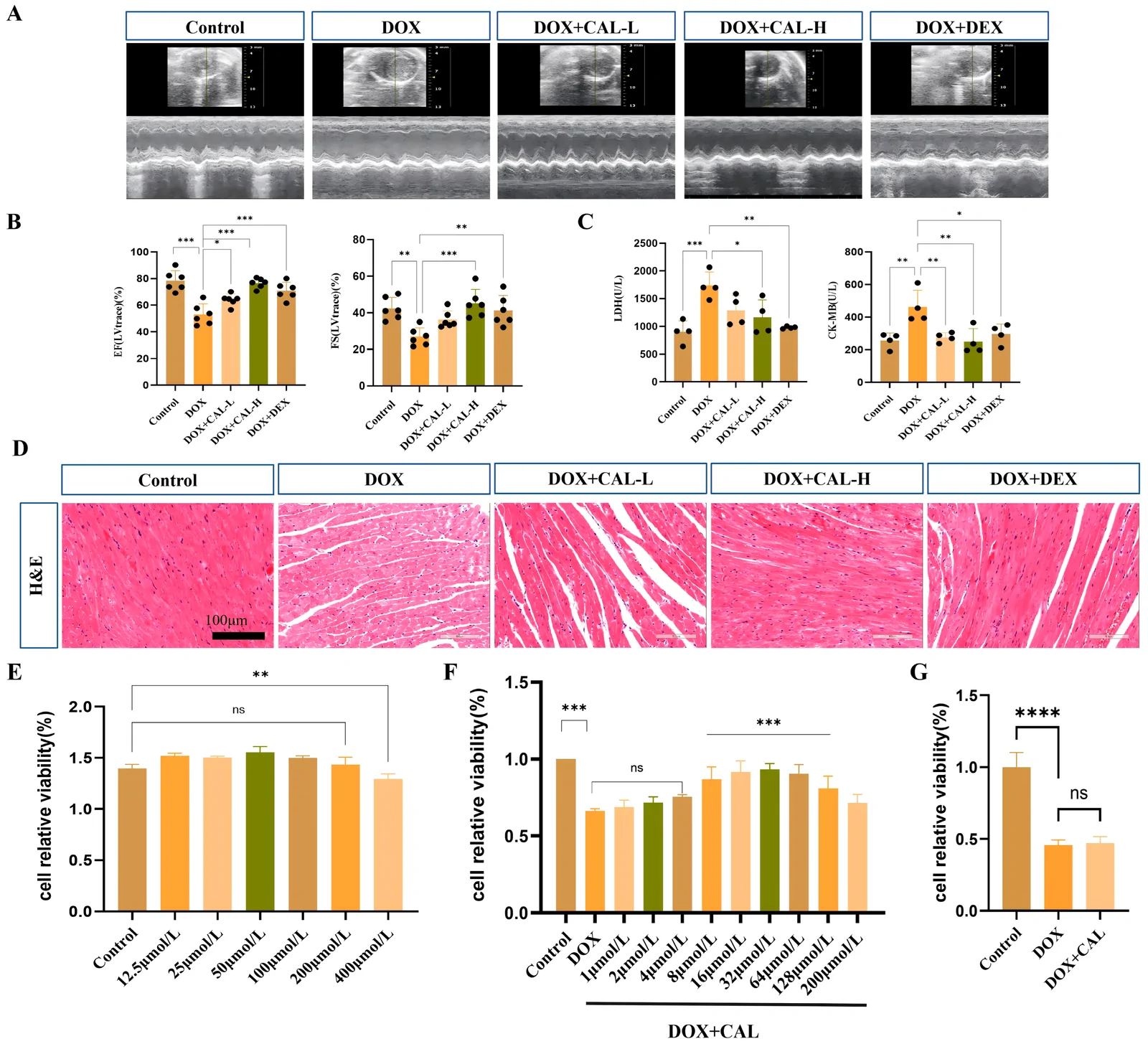
Calycosin improves cardiac dysfunction in a DIC mouse model. (**A**) Echocardiographic evaluation (*n* = 6 mice per group). (**B**) Quantitative analysis of cardiac function parameters (*n* = 6 mice per group). (**C**) Measurement of serum biomarkers of myocardial injury (*n* = 4 mice per group). (**D**) Representative histological images obtained by H&E staining (scale bar = 100 μm). (**E**) Viability of H9c2 cells treated with different concentrations of CAL (*n* = 6 independent biological experiments). (**F**) Effects of CAL and DOX on H9c2 cell viability (*n* = 6 independent biological experiments). (**G**) Viability of 4T1 breast cancer cells following DOX treatment with or without CAL (*n* = 6 independent biological experiments). Data represent mean ± SD. ns, not significant; * *p* < 0.05, ** *p* < 0.01, *** *p* < 0.001, and **** *p* < 0.0001.

To investigate the cardioprotective effects of CAL, a murine model of DIC was first established. Mice treated with DOX (6 mg/kg) exhibited a profound decline in cardiac performance, with the EF and FS decreasing by 47.59% and 57.54%, respectively (Table 1). Oral administration of CAL dose-dependently restored these parameters and significantly decreased serum levels of myocardial injury markers, including CK-MB and LDH (Figure 1B,C). Histopathological analysis using H&E staining further demonstrated that CAL alleviated DOX-induced myocardial structural abnormalities, such as myofibrillar disorganization and interstitial expansion, indicating its protective role in maintaining cardiac integrity (Figure 1D).

Concurrently, we validated the protective effects of CAL against DOX-induced myocardial injury in vitro. CAL demonstrated no cytotoxicity in H9c2 cardiomyocytes across a concentration range of 3.125–200 µM and significantly enhanced the proliferative capacity of H9c2 cells at concentrations ranging from 8–200 µM (Figure 1E,F). Furthermore, to assess whether CAL interferes with the antitumor activity of DOX, a CCK-8 assay was conducted in 4T1 breast cancer cells. The results indicated that CAL did not affect the anticancer efficacy of DOX in 4T1 cells (Figure 1G).

### 3.2. Transcriptomic Profiling Reveals Mitochondrial Energy Metabolism and Ferroptosis as Potential Cardioprotective Mechanisms of Calycosin in DIC

**Figure 2:**
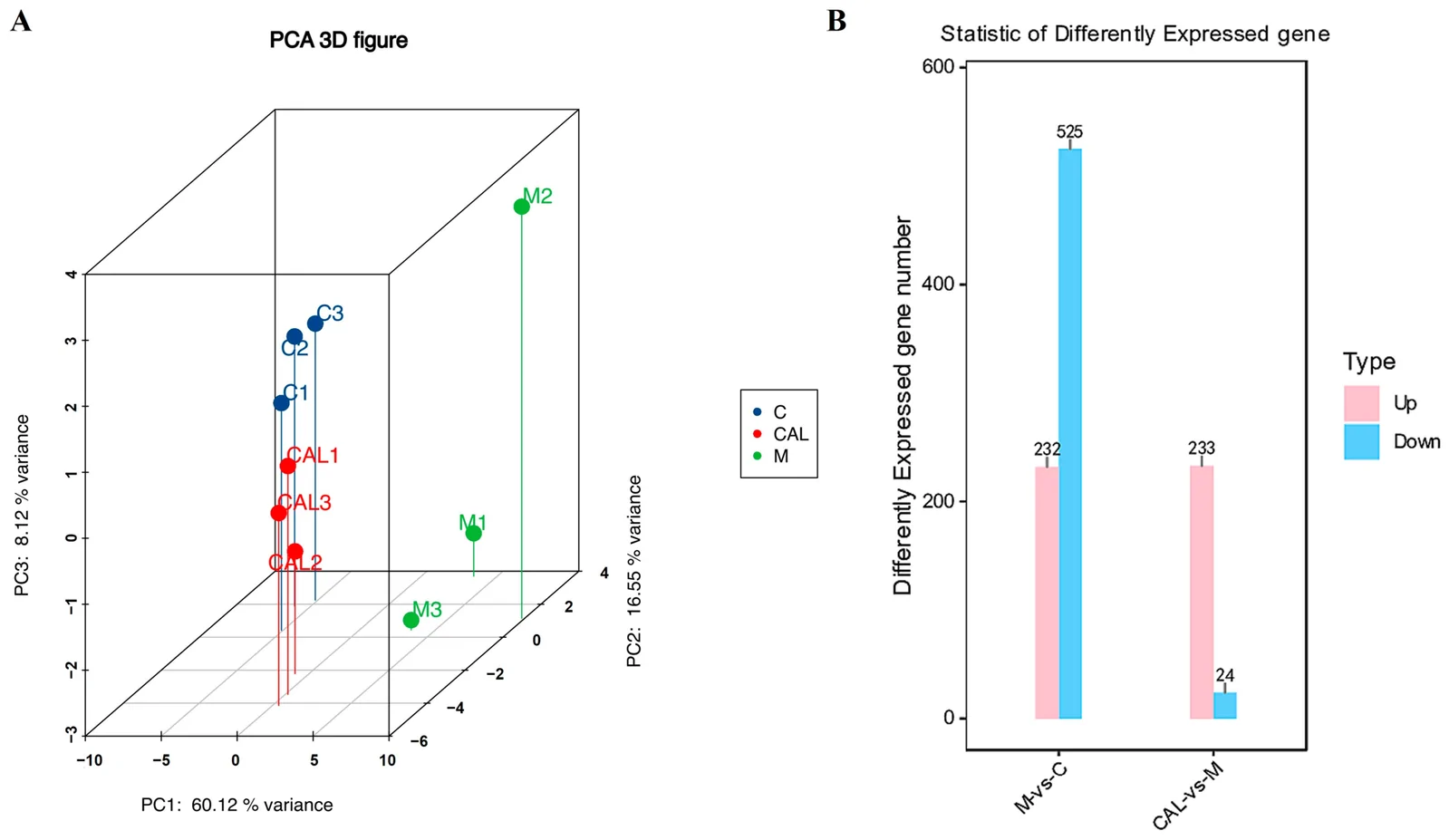

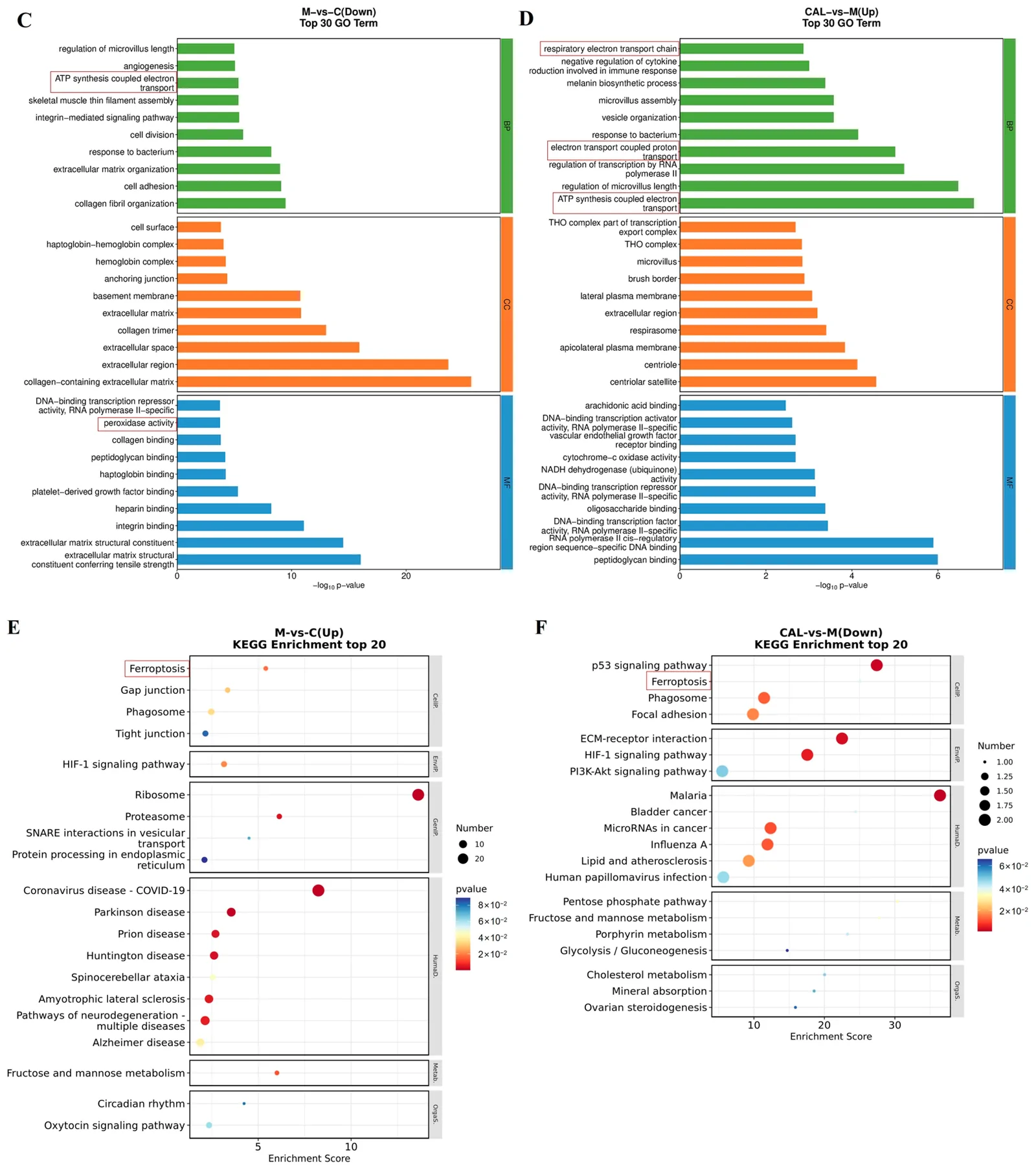
Transcriptomic analysis of mouse hearts revealed that calycosin attenuated DIC by restoring mitochondrial energy metabolism and suppressing ferroptosis. (**A**) A three-dimensional PCA plot illustrating the clustering patterns among the Control group (**C**), the DOX-treated model group (M), and the DOX + CAL group (CAL), (*n* = 3 mice per group). The same abbreviations are used throughout Figure 2. (**B**) Bar chart showing the number of differentially expressed genes (DEGs) in the M-vs-C and CAL-vs-M comparisons. (**C**) GO enrichment analysis of downregulated genes in the M-vs-C comparison. (**D**) GO enrichment analysis of upregulated genes in the CAL-vs-M comparison. (**E**) KEGG pathway enrichment analysis of upregulated genes in the M-vs-C comparison. (**F**) KEGG pathway enrichment analysis of downregulated genes in the CAL-vs-M comparison. Terms highlighted in red represent the primary focus of this study.

To identify the molecular drivers behind the protective effects of CAL, we performed transcriptomic sequencing on cardiac tissues. Principal component analysis (PCA) revealed distinct clustering among the Control, DOX, and DOX + CAL groups (Figure 2A). The numbers of upregulated and downregulated DEGs in the DOX-versus-Control and CAL-versus-DOX comparisons are summarized in Figure 2B. Differential expression analysis and functional enrichment revealed that CAL treatment significantly reversed DOX-induced impairment of mitochondrial energy metabolism and pathological activation of ferroptosis. Specifically, CAL upregulated genes enriched in “ATP synthesis-coupled electron transport” and the “respiratory electron transport chain” (Figure 2C,D), while KEGG analysis confirmed its potent modulation of the ferroptosis pathway (Figure 2E,F).

### 3.3. Calycosin Preserves Mitochondrial Ultrastructure and Redox Homeostasis

**Figure 3:**
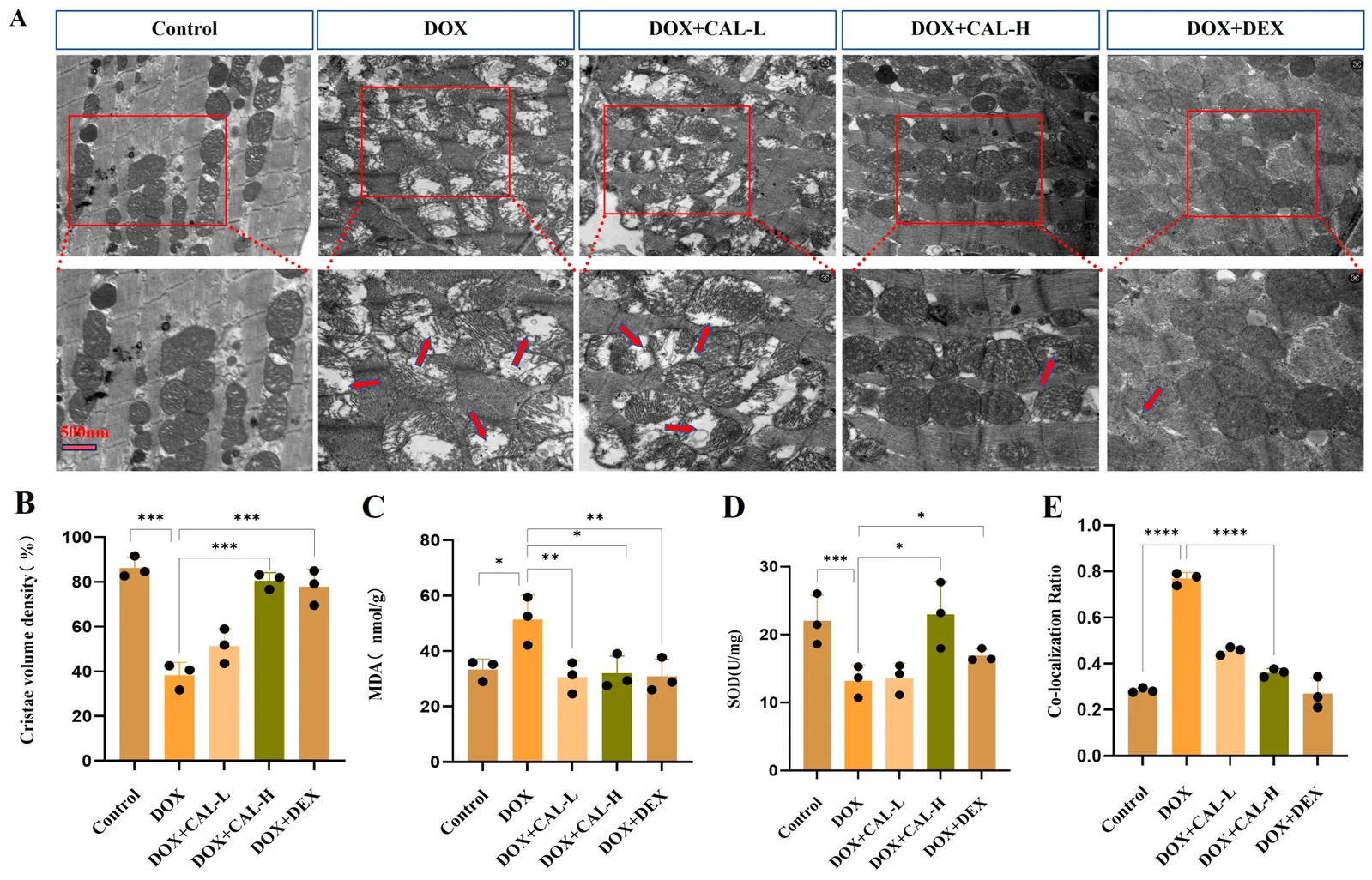

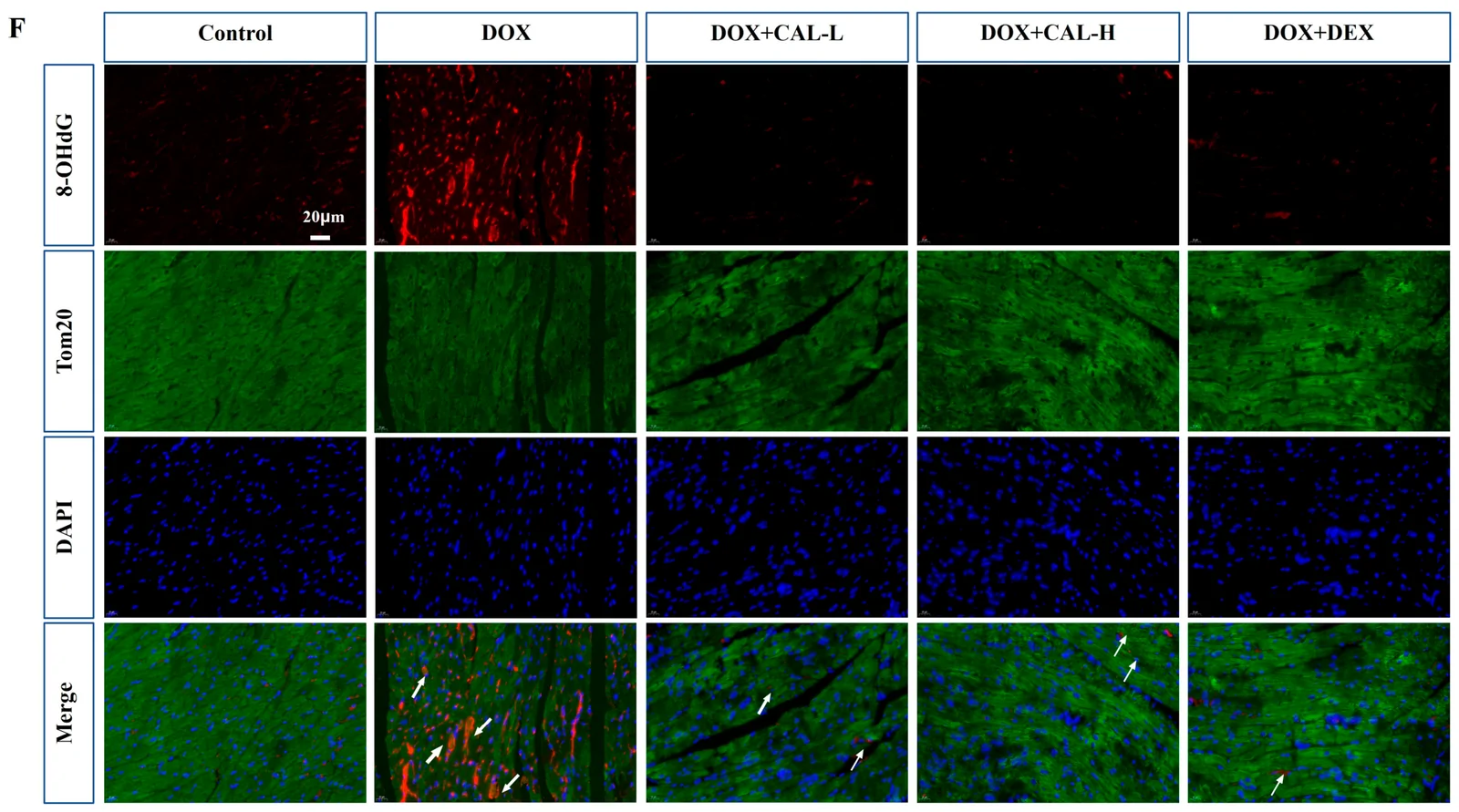
Calycosin ameliorates DOX-mediated mitochondrial dysfunction in vivo. (**A**) TEM analysis showing calycosin treatment preserved mitochondrial architecture (*n* = 3 mice per group). (**B**) Quantitative analysis of mitochondrial cristae volume density in TEM images (*n* = 3 mice per group). (**C**,**D**) Cardiac oxidative stress parameters (lipid peroxidation marker MDA and antioxidant enzyme SOD; *n* = 3 mice per group). (**E**) Quantitative colocalization analysis of 8-OHdG and Tom20 using Pearson’s correlation coefficient (*n* = 3 mice per group). (**F**) Representative immunofluorescence images illustrating DNA damage evaluated by 8-OHdG staining (scale bar = 20 μm; *n* = 3 mice per group). Data are presented as mean ± SD. * *p* < 0.05, ** *p* < 0.01, *** *p* < 0.001, and **** *p* < 0.0001 compared with DOX group.

Guided by the transcriptomic findings, we subsequently assessed mitochondrial integrity in cardiac tissues. Transmission electron microscopy (TEM) of the myocardium showed that DOX caused severe mitochondrial vacuolization and cristae destruction, effects that were markedly attenuated by CAL treatment (Figure 3A,B). Furthermore, CAL treatment significantly bolstered the heart’s antioxidant capacity, as evidenced by restored superoxide dismutase (SOD) activity and a significant reduction in malondialdehyde (MDA) content, a marker of lipid peroxidation (Figure 3C,D). Immunofluorescence analysis revealed that calycosin significantly mitigated mitochondrial DNA (mtDNA) damage, as evidenced by reduced levels of 8-hydroxydeoxyguanosine (8-OHdG) staining in doxorubicin-treated murine models (Figure 3E,F). These results suggest that CAL stabilizes the mitochondrial environment against DOX-induced oxidative stress.

### 3.4. Molecular Docking Identifies NRF2/NRF1 as Potential Targets of Calycosin in DIC

**Figure 4:**
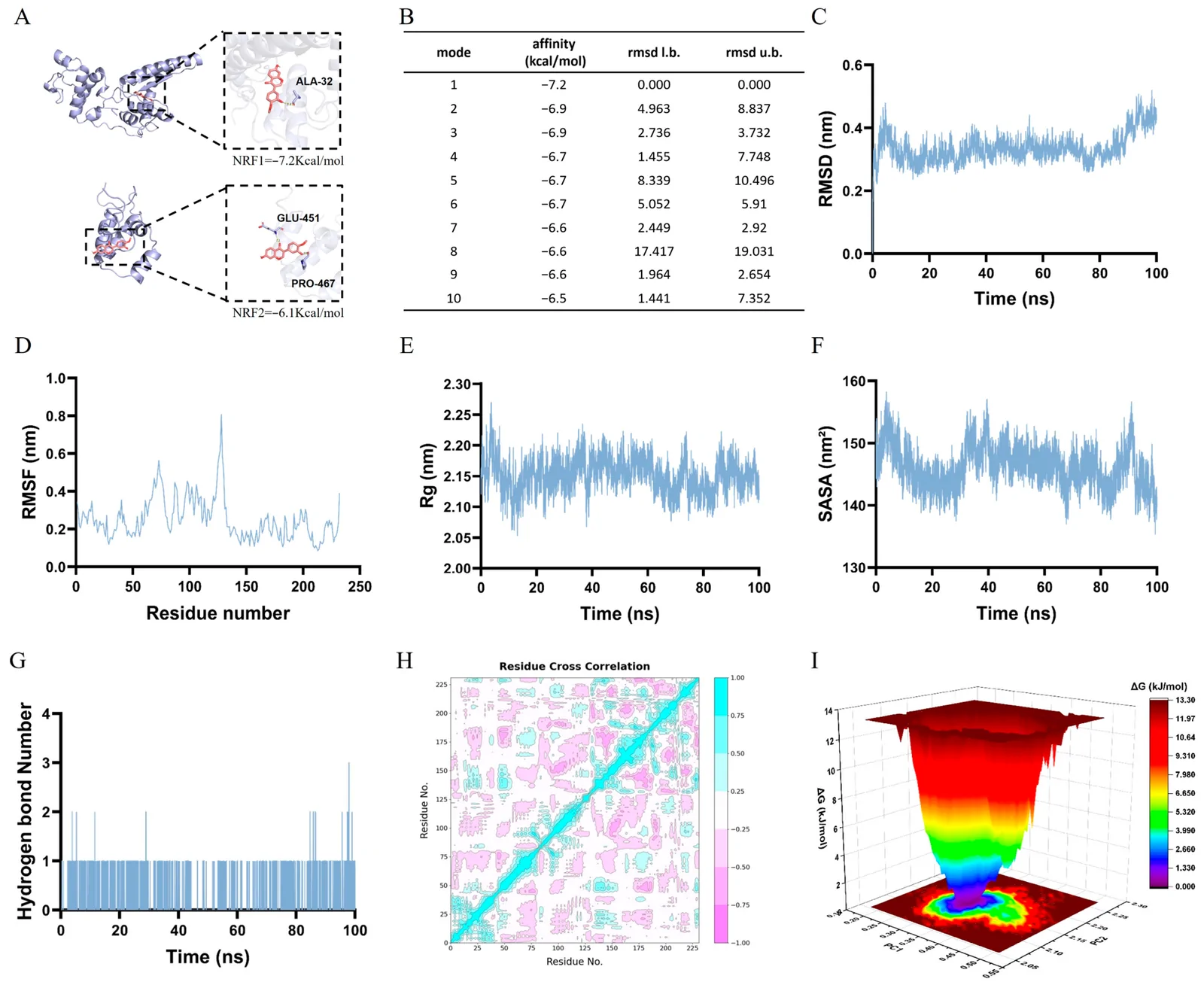
Molecular docking and molecular dynamics simulation analysis of the calycosin-NRF1 complex. (**A**) Schematic illustration of the docking conformations of calycosin within the binding pockets of NRF1 and NRF2. (**B**) Binding energy profile of calycosin with NRF1. (**C**) RMSD analysis of the calycosin-NRF1 complex. (**D**) RMSF profile of NRF1. (**E**) Rg of the NRF1 backbone. (**F**) SASA analysis. (**G**) Hydrogen bond interactions within the complex. (**H**) Hydrogen bond interactions within the complex. (**I**) FEL of the calycosin-NRF1 complex in 3D.

Given that the NRF2/NRF1 signaling axis serves as a classical upstream regulator of both redox homeostasis [26] and mitochondrial bioenergetics [27], we hypothesized that CAL might directly target these transcription factors. Molecular docking analysis was performed, revealing strong binding affinities between CAL and NRF1 (−7.2 kcal/mol) as well as NRF2 (−6.1 kcal/mol). To further assess the structural stability of the NRF1–CAL complex, we employed a range of molecular dynamics simulation techniques, including root mean square deviation (RMSD), root mean square fluctuation (RMSF), radius of gyration (Rg), solvent-accessible surface area (SASA), hydrogen bond analysis, and free energy landscape (FEL) (Figure 4C–I). The combined docking and simulation results indicated that CAL forms a stable complex with NRF1, primarily through hydrogen bonding and other intermolecular interactions. These findings suggested that CAL may act as a direct ligand or stabilizer for the NRF2/NRF1 axis, leading us to further investigate the role of this signaling pathway in CAL’s cardioprotective effects.

### 3.5. Validation of NRF2/NRF1-TFAM Axis Activation and Nuclear Translocation

**Figure 5:**
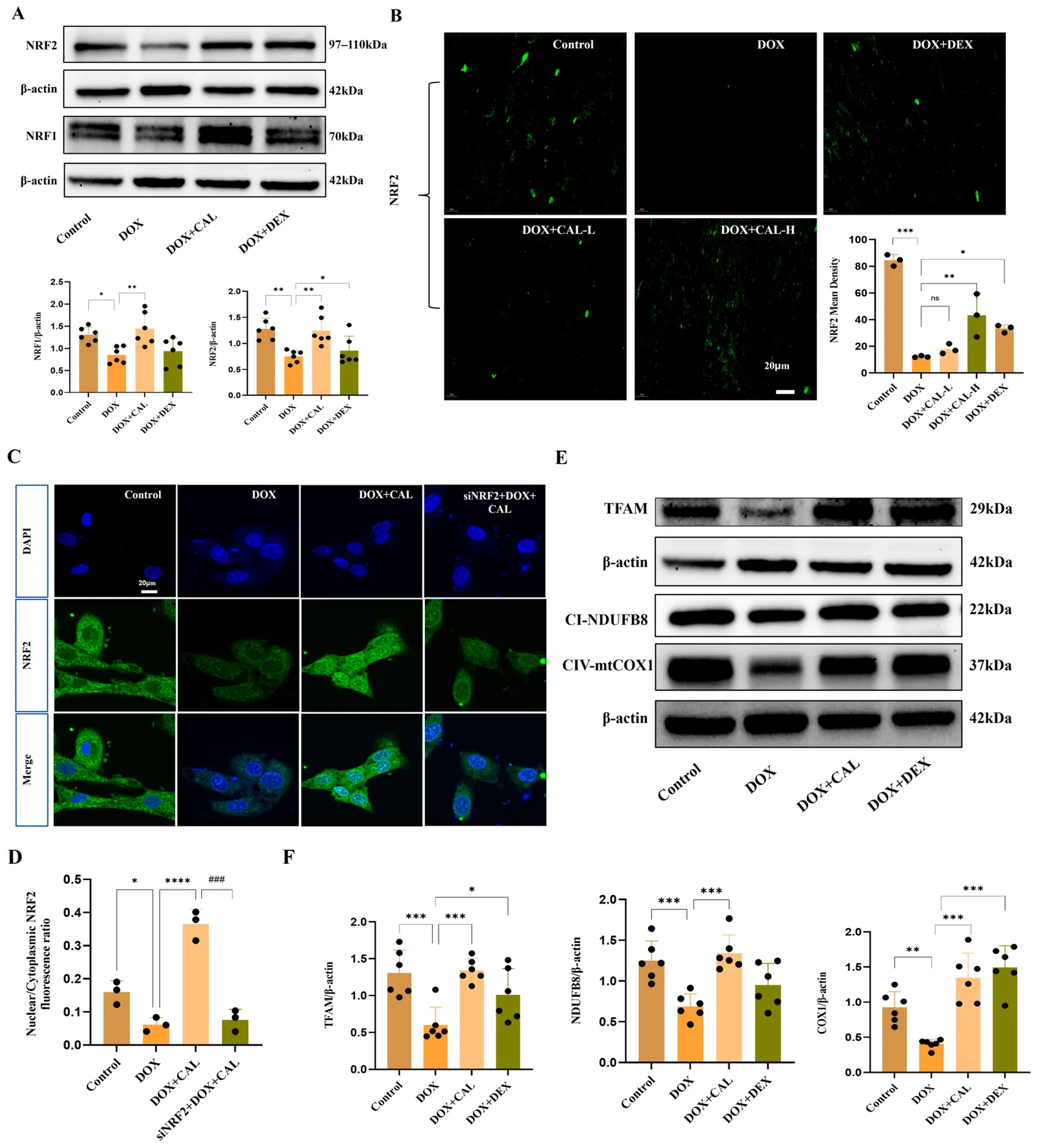
Calycosin activates the NRF2/NRF1-TFAM signaling axis and promotes NRF2 nuclear translocation. (**A**) Representative Western blot and quantitative analysis of NRF2 and NRF1 protein expression in cardiac tissues (*n* = 6 mice per group). (**B**–**D**) Immunofluorescence analysis of NRF2 localization in cardiac tissue sections (*n* = 3 mice per group) and H9c2 cardiomyocytes (scale bar = 20 μm; n = 3 independent biological experiments). (**E**,**F**) Western blot analysis of TFAM and mitochondrial respiratory chain subunits (NDUFB8 and COX1; *n* = 6 mice per group). ns, not significant; * *p* < 0.05, ** *p* < 0.01, *** *p* < 0.001, and **** *p* < 0.0001 vs. DOX group; ^###^ *p* < 0.001 vs. the DOX + CAL group.

Based on the results obtained from molecular docking and dynamics simulations, we further investigated the activation of the NRF2/NRF1 signaling axis, which served as the master regulator of mitochondrial biogenesis. In vivo Western blotting confirmed that CAL treatment significantly reversed the DOX-induced downregulation of NRF2 and NRF1 (Figure 5A). Furthermore, immunofluorescence in H9c2 cardiomyocytes demonstrated that CAL promoted the nuclear translocation of NRF2, providing evidence for this event as the upstream signaling stimulus (Figure 5B–D).

The structural and functional integrity of mitochondria ultimately depends on the stable expression and transcription of the mitochondrial genome. Mitochondrial transcription factor A (TFAM) is a critical downstream effector of NRF1 that not only drives the transcription of mitochondrial DNA (mtDNA) but also packages mtDNA into nucleoids to protect it from oxidative damage. Given that DOX targets and disrupts mtDNA, we investigated whether the CAL-induced activation of NRF1 extended to the stabilization of TFAM. Notably, CAL treatment effectively restored TFAM protein levels, which was accompanied by a robust increase in downstream mitochondrial respiratory chain subunits, including NDUFB8 and COX1 (Figure 5E,F). These results validate the complete NRF2/NRF1-TFAM signaling cascade predicted by our transcriptomic analysis and suggest that CAL safeguards mitochondrial bioenergetics by maintaining the mtDNA-regulatory machinery.

### 3.6. CAL Maintains Mitochondrial Iron Homeostasis and Biogenesis via the NRF2-Mediated NRF1/TFAM Axis

**Figure 6:**
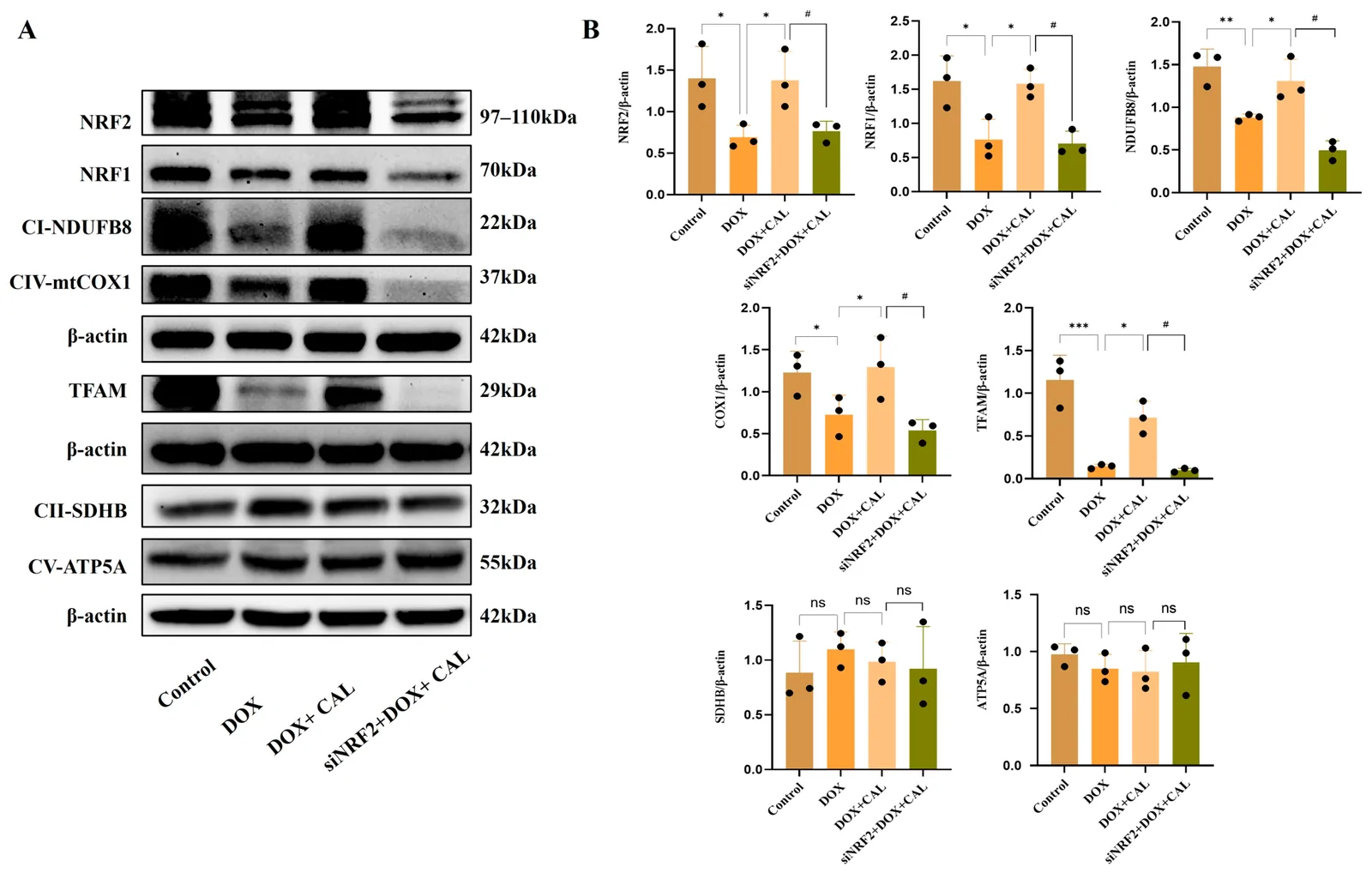

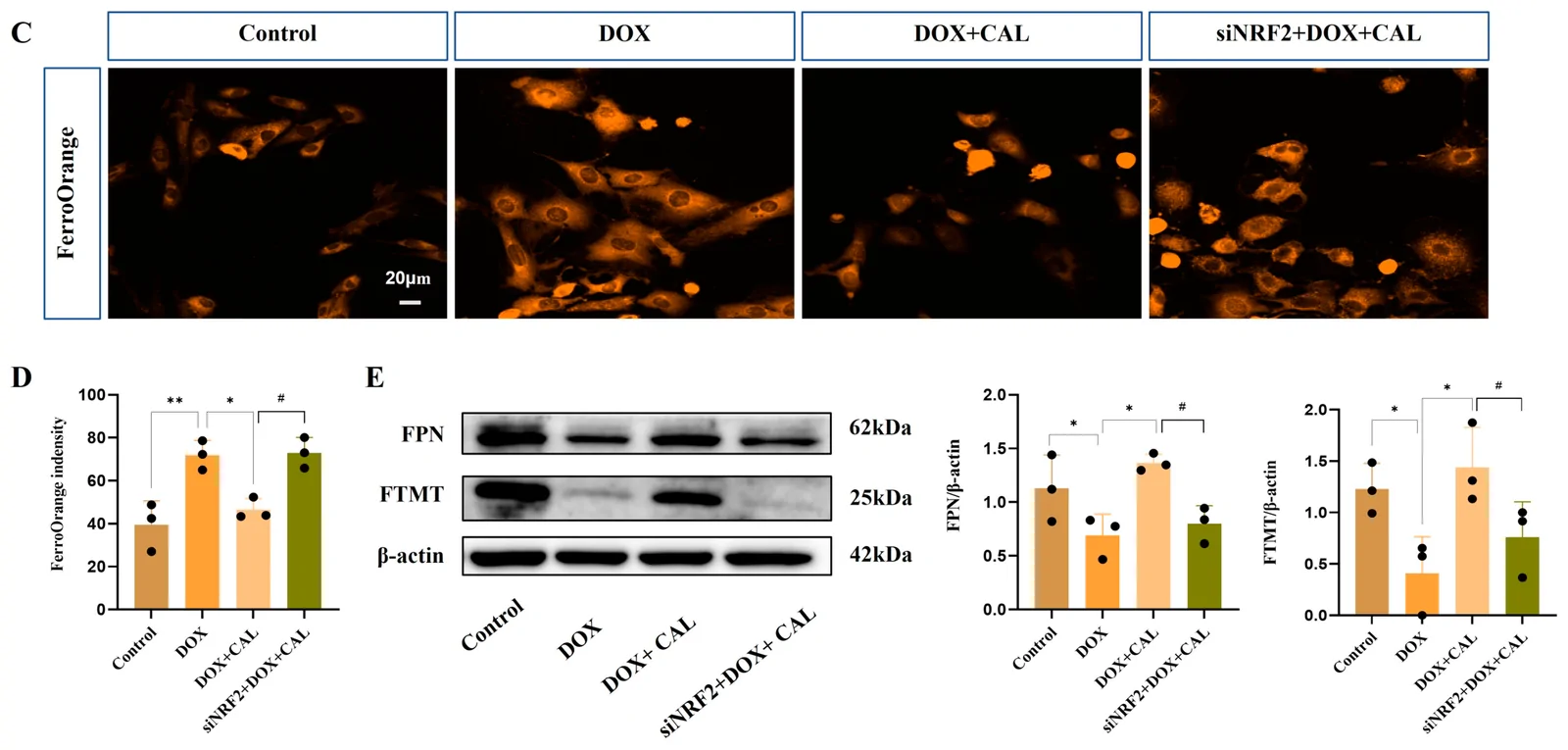
Calycosin preserves mitochondrial biogenesis and suppresses ferroptosis via the NRF2-mediated NRF1/TFAM axis. (**A**,**B**) Assessment of mitochondrial biogenesis. NRF2 knockdown abolished the protective effects of CAL on the protein levels of NRF1, TFAM, COX1, and NDUFB8, while no significant changes were observed in SDHB or ATP5A (*n* = 3 independent biological experiments). (**C**,**D**) NRF2 knockdown abolished CAL-mediated ferroptosis suppression (*n* = 3 independent biological experiments). (**E**) NRF2 knockdown abolished CAL-mediated iron regulation. Following siRNA-mediated silencing of NRF2, CAL failed to upregulate the expression of FTMT and FPN (*n* = 3 independent biological experiments). * *p* < 0.05, ** *p* < 0.01, *** *p* < 0.001 vs. DOX group; ^#^ *p* < 0.05 vs. DOX + CAL group, ns: not significant.

Beyond restoring mitochondrial biogenesis (Figure 6A,B), CAL treatment significantly suppressed DOX-induced ferroptosis by targeting mitochondrial iron overload. We observed that CAL markedly upregulated mitochondrial ferritin (FTMT) and the iron exporter Ferroportin (FPN), effectively buffering the labile Fe pool and preventing lipid peroxidation. Crucially, knockdown of NRF2 using a rat-specific siRNA in H9c2 cells completely abolished CAL’s ability to regulate FTMT/FPN and reduce iron accumulation (Figure 6C–E). These results identify NRF2 as the indispensable node through which CAL orchestrates mitochondrial iron homeostasis to counteract ferroptotic damage. However, we reasoned that preventing iron-induced lipid peroxidation alone may not suffice if the underlying mitochondrial architecture remains compromised. Given that CAL partially restored mitochondrial function, including TFAM and the respiratory-chain proteins NDUFB8 and COX1, we next examined whether these effects were dependent on downstream NRF1 signaling.

### 3.7. NRF1 Serves as the Essential Link Between NRF2 Signaling and Mitochondrial Genomic Integrity and Function

**Figure 7:**
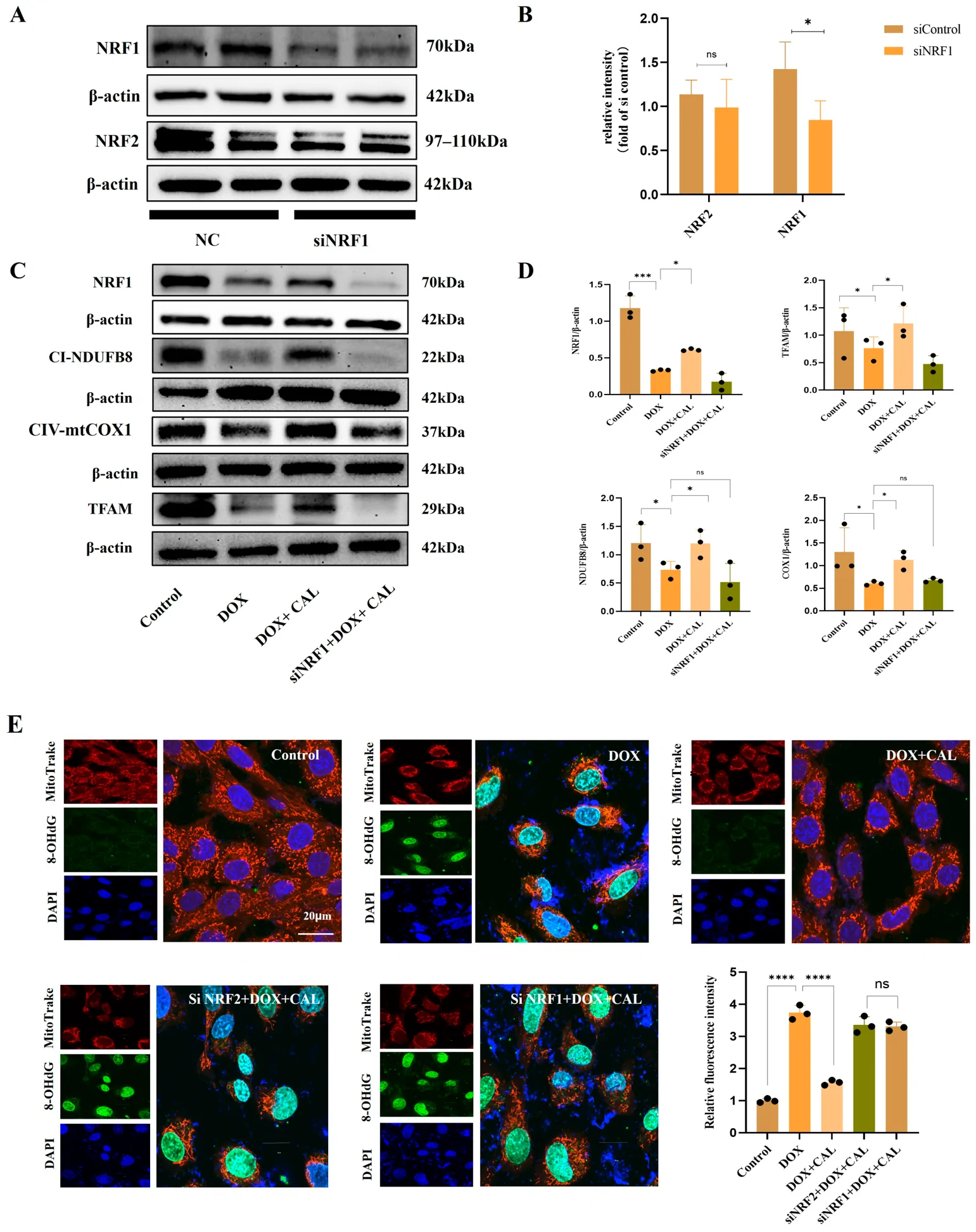

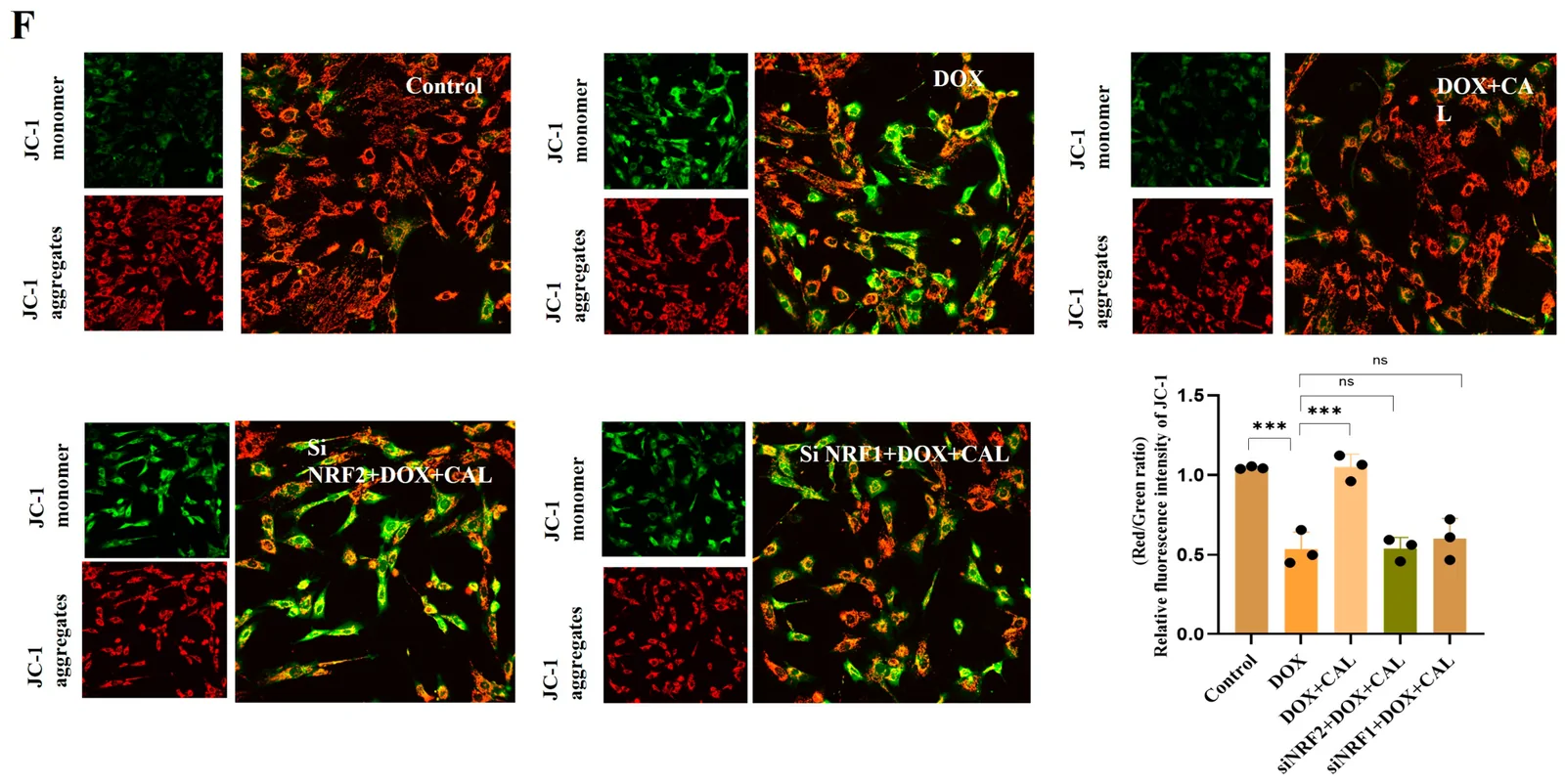
NRF1 functions as the essential downstream effector linking NRF2 signaling to mitochondrial genomic integrity and function. (**A**–**D**) Effects of NRF1 silencing on CAL-mediated mitochondrial protection. siRNA-mediated knockdown of NRF1 did not alter NRF2 protein levels, but completely abrogated CAL-induced upregulation of TFAM, COX1, and NDUFB8 (*n* = 3 independent biological experiments). (**E**,**F**) NRF1 knockdown abolishes CAL-mediated preservation of mitochondrial genome integrity and function. Loss of NRF1 abrogated CAL’s protective effect against DOX-induced mtDNA damage, as reflected by elevated 8-OHdG levels and resulted in a reduction in mitochondrial membrane potential (Δψm; *n* = 3 independent biological experiments). ns, not significant; * *p* < 0.05, *** *p* < 0.001, and **** *p* < 0.0001 vs. DOX group.

To further dissect this regulatory hierarchy, we focused on NRF1 as the critical bridge translating NRF2 activation into functional mitochondrial recovery. Our results demonstrate that while NRF2 initiates the defense program, NRF1 is the indispensable “structural effector” required to stabilize the mitochondrial genome. siRNA-mediated silencing of NRF1 did not affect NRF2 levels, but completely abrogated CAL’s ability to restore TFAM and respiratory subunits (NDUFB8, COX1) (Figure 7A–D).

Crucially, NRF1 knockdown also neutralized CAL’s protective effects on mitochondrial DNA (mtDNA) integrity, evidenced by a surge in 8-OHdG levels and the subsequent collapse of mitochondrial membrane potential (Δψm) (Figure 7E,F). These results demonstrate that while NRF2 initiates the defense program, NRF1 is the indispensable “structural effector” required to stabilize the mitochondrial genome and bioenergetic machinery. This highlights a novel dependency where NRF2-mediated iron control must be coupled with NRF1-driven structural repair to fully rescue the cell from energy collapse and ferroptotic death.

Together, these data demonstrate that NRF2-driven iron homeostasis and NRF1-mediated structural repair represent two mechanistically distinct yet functionally interdependent modules, both of which are required for CAL to confer full cardioprotection against ferroptosis and bioenergetic failure.

### 3.8. In Vivo Inhibition of NRF2 Abolishes the Therapeutic Efficacy of CAL Against DIC

**Figure 8:**
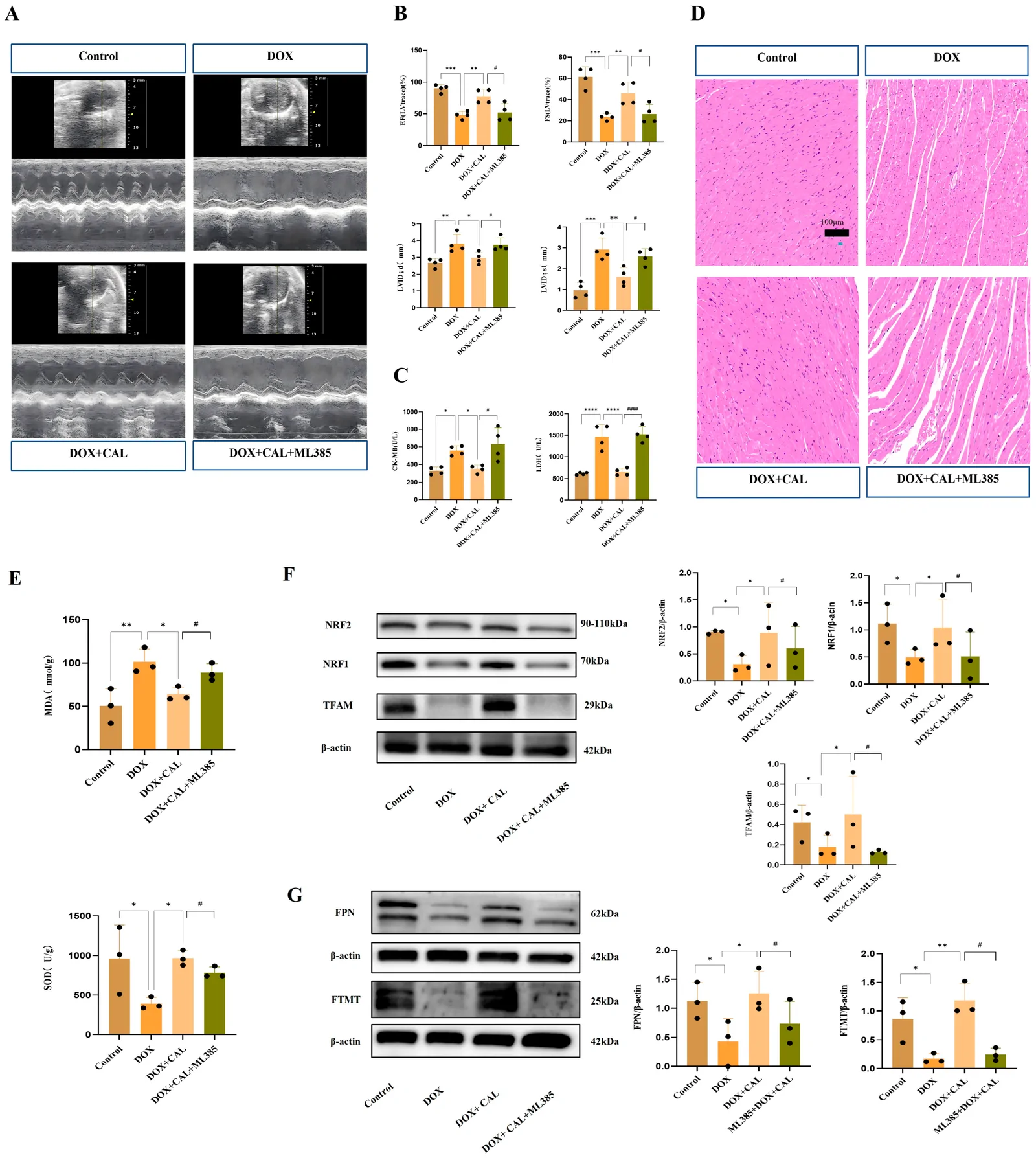
Pharmacological inhibition of NRF2 abolishes CAL-mediated cardioprotection, mitochondrial recovery, and iron homeostasis in vivo. (**A**,**B**) Echocardiographic assessment of cardiac function. CAL treatment significantly improved DOX-impaired Ejection Fraction (EF) and Fractional Shortening (FS), whereas co-administration with the NRF2 inhibitor ML385 completely negated this functional recovery (*n* = 6 mice per group). (**C**) Serum levels of cardiac injury markers. CAL significantly reduced DOX-induced elevations in CK-MB and LDH, effects that were effectively abrogated by ML385 co-treatment (*n* = 4 mice per group). (**D**) Representative H&E staining of cardiac tissue sections. CAL ameliorated DOX-induced myofibril disarray and inflammatory cell infiltration; however, this structural preservation was largely abolished upon NRF2 inhibition. (**E**) Cardiac oxidative stress parameters. CAL suppressed DOX-induced MDA elevation and restored SOD activity, both of which were reversed by ML385 co-administration (*n* = 3 mice per group). (**F**,**G**) Western blot analysis of NRF2/NRF1-TFAM axis and iron metabolism proteins. ML385-mediated inhibition of NRF2 abolished CAL-induced upregulation of NRF1, TFAM, COX1, and NDUFB8 (**F**), as well as FTMT and FPN (**G**), confirming that NRF2 functions as the essential master switch orchestrating both mitochondrial biogenesis and iron homeostasis (*n* = 3 mice per group). * *p* < 0.05, ** *p* < 0.01, *** *p* < 0.001, and **** *p* < 0.0001 vs. DOX group; ^#^ *p* < 0.05, ^####^ *p* < 0.001 vs. DOX + CAL group.

To further validate the physiological relevance of NRF2 signaling, we employed the NRF2 inhibitor ML385 in a murine model. While CAL treatment alone significantly improved cardiac contractility, co-administration with ML385 effectively negated the recovery of EF and FS (Figure 8A,B and Table 2). While CAL treatment alone significantly reduced serum levels of cardiac injury markers CK-MB and LDH, co-administration with ML385 effectively abrogated this protective effect, resulting in a marked elevation of both markers (Figure 8C). Furthermore, HE staining revealed that CAL ameliorated DOX-induced disorganization of myocardial myofibrils and inflammatory cell infiltration; however, this structural preservation was largely abolished upon ML385 treatment (Figure 8D). Moreover, the antioxidant efficacy of CAL was also NRF2-dependent, as co-administration with ML385 reversed CAL-mediated suppression of MDA and restoration of SOD activity (Figure 8E). At the molecular level, Western blot analysis of heart tissue revealed that ML385-mediated inhibition of NRF2 suppressed the CAL-induced activation of the NRF2/NRF1-TFAM axis and paralyzed the regulation of iron metabolism proteins, specifically FTMT and FPN (Figure 8F,G). This “loss-of-function” evidence in vivo confirms that NRF2 is the essential master switch through which CAL orchestrates both mitochondrial biogenesis and iron homeostasis to rescue the heart from DOX-induced injury.

## 4. Discussion

**Figure 9:**
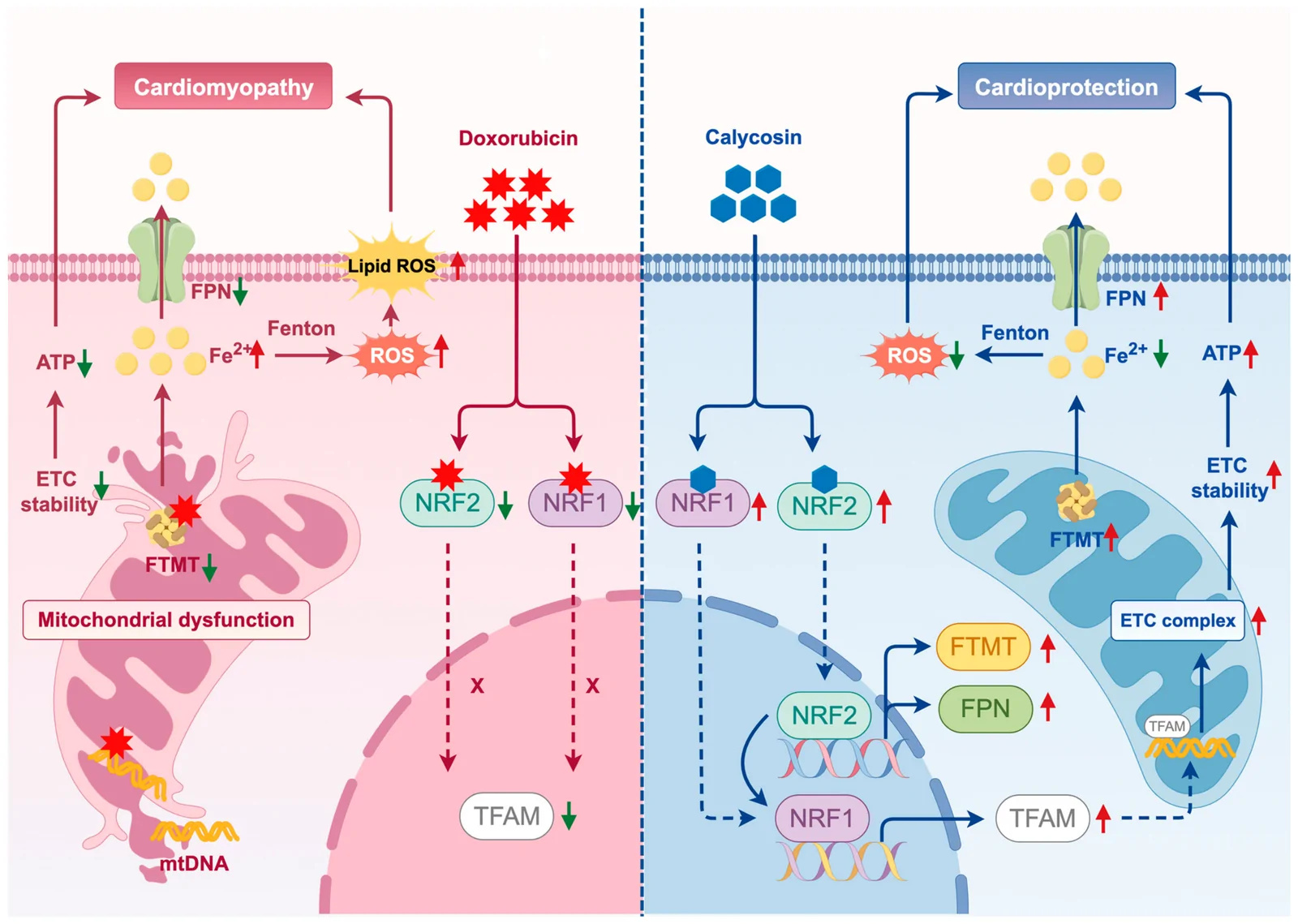
Schematic diagram illustrating that calycosin exerts cardioprotective effects via the NRF1/NRF2-TFAM signaling axis. Calycosin restores both NRF2 and NRF1 expression and promotes NRF2 activation. Activated NRF2 contributes to mitochondrial iron homeostasis through regulation of FTMT and FPN and further supports downstream NRF1–TFAM signaling. In parallel, calycosin-mediated restoration of NRF1 promotes TFAM and mitochondrial respiratory-chain components, including COX1 and NDUFB8, thereby contributing to the maintenance of mitochondrial genomic integrity and function. Through NRF2-mediated regulation of mitochondrial iron homeostasis and NRF1–TFAM-dependent maintenance of mitochondrial function, calycosin attenuates DOX-induced oxidative stress, ferroptosis, and myocardial injury.

Our study elucidates a novel and coordinated mechanism by which CAL protects against DIC. We demonstrated that CAL exerts its cardioprotective effect primarily by activating the integrated NRF2/NRF1-TFAM signaling axis, which functions as a dual-regulatory hub to concurrently preserve mitochondrial genomic integrity and restore iron homeostasis (Figure 9). This simultaneous targeting of both the bioenergetic foundation and the ferroptotic driver of DIC represents a significant advance over single-pathway interventions and provides a comprehensive mechanistic basis for the efficacy of CAL.

Transcriptomic analysis provided the initial systems-level insight into the mechanism of action of CAL. Treatment with CAL significantly reversed DOX-induced dysregulation of genes involved in mitochondrial energy metabolism and ferroptosis pathways, suggesting that these two pathological processes may be coordinately regulated rather than independently targeted. The common upstream regulatory node linking mitochondrial bioenergetics and iron-dependent cell death was subsequently investigated.

Given that ferroptosis is primarily governed by redox and iron-handling pathways, whereas mitochondrial energy metabolism is tightly regulated by transcriptional programs controlling mitochondrial biogenesis, these findings directed our focus to two key upstream regulators: NRF2, a central mediator of antioxidant defense and ferroptosis suppression [28,29], and NRF1, a master regulator of mitochondrial transcription and bioenergetic maintenance [30]. Thus, the NRF2/NRF1 axis provides a biologically plausible framework linking the two major transcriptomic signatures observed in our study.

To further explore whether CAL could potentially regulate these upstream targets, molecular docking and molecular dynamics simulations were performed. The results indicated favorable binding of CAL to both NRF2 and NRF1 and supported the stability of the protein–ligand complex. These findings provide supportive structural evidence that CAL may interact with this regulatory axis, thereby strengthening the rationale for subsequent mechanistic validation.

A key innovative finding of our study is the NRF2-dependent reprogramming of cardiac iron metabolism. Although ferroptosis has been recognized in DIC [31], most inhibitors target the canonical glutathione–GPX4 axis [32,33,34]. In contrast, we found that CAL uniquely upregulates mitochondrial ferritin (FTMT) and the iron exporter ferroportin (FPN), and this regulatory effect was markedly abolished by NRF2 knockdown, highlighting the essential role of NRF2 in this process. This action selectively reduces the labile iron pool at its primary pathological source—the mitochondria—where Fenton chemistry is most active [35,36]. By enhancing iron sequestration (FTMT) and efflux (FPN), CAL directly quenches the reactive iron catalyst, offering a more precise therapeutic strategy than antioxidant scavenging.

Preventing iron overload alone is insufficient if the underlying bioenergetic architecture remains compromised. We established that NRF1 serves as the indispensable structural effector that translates NRF2 signaling into mitochondrial protection. Notably, NRF2 silencing reduced NRF1 protein expression, whereas NRF1 silencing did not substantially alter NRF2 levels. This asymmetric response suggests a hierarchical relationship in which NRF2 functions upstream of NRF1 under our experimental conditions, rather than a mutually dependent regulatory loop. However, whether NRF2 directly regulates NRF1 at the transcriptional level remains to be determined. While NRF2 initiates the antioxidant response and iron control, the maintenance of mitochondrial DNA (mtDNA) integrity and respiratory chain (NDUFB8 and COX1) is strictly dependent on the NRF1-TFAM axis. Notably, NRF1 knockdown abolished CAL’s protective effects even in the presence of NRF2. This underscores a critical functional dependency: mitigating ferroptosis provides only a temporary protection unless the underlying bioenergetic infrastructure is simultaneously repaired.

From a clinical perspective, unlike dexrazoxane, whose iron-chelating mechanism carries systemic risks, CAL may offer a safer alternative by acting through endogenous transcriptional programs to restore cellular homeostasis. Additionally, our in vitro study confirmed that CAL did not impair the antitumor efficacy of DOX. Collectively, our findings identify the NRF2/NRF1-TFAM axis as a previously underappreciated, integrative therapeutic target for DIC.

Nonetheless, several limitations warrant further investigation. While molecular docking predicted high binding affinities for NRF2 and NRF1, biophysical techniques such as surface plasmon resonance are required to confirm direct ligand–protein interactions and characterize the precise binding kinetics. In addition, H9c2 cells are undifferentiated rat cardiomyoblasts and may not fully recapitulate the physiological characteristics of mature cardiomyocytes. Our study also focused on immunocompetent murine models; therefore, further validation using primary or human iPSC-derived cardiomyocytes and tumor-bearing animal models is needed to strengthen the clinical translatability of our findings.

## 5. Conclusions

In conclusion, this study identifies the NRF2/NRF1-TFAM axis as the primary therapeutic target through which calycosin mitigates DIC. Calycosin exerts its effects via a dual-action mechanism: NRF2-mediated restoration of mitochondrial iron homeostasis, specifically through FTMT and FPN upregulation, and NRF1-driven stabilization of the mitochondrial genome and respiratory chain. Our findings establish a critical regulatory hierarchy where iron control is functionally coupled with structural biogenesis. This iron–respiratory crosstalk is essential for maintaining cardiomyocyte viability and preventing the energy collapse that drives DIC. Ultimately, these results provide a robust molecular rationale for developing calycosin as a safer alternative to current cardioprotective agents, offering a novel strategy to safeguard cardiac function during anthracycline chemotherapy.

## Figures and Tables

**Table 1:** The results of echocardiography in each group of mice (Mean ± SD; n = 6 mice per group).

Group	EF (%)	FS (%)
Control	78.34 ± 7.576	42.36 ± 6.032
DOX	53.08 ± 7.851 ***	26.89 ± 4.971 **
CAL-L	63.97 ± 4.410 *	39.81 ± 7.874 *
CAL-H	76.58 ± 3.685 ***	45.13 ± 7.699 ***
DEX	70.86 ± 6.475 ***	41.17 ± 8.231 **

In contrast to the group treated with DOX, * *p* < 0.05, ** *p* < 0.01, *** *p* < 0.001.

**Table 2:** Data are expressed as mean ± SD (*n* = 6 mice per group).

Group	LVEF (%)	LVFS (%)	LVEDD (mm)	LVESD (mm)
Control	89.87 ± 5.976	61.31 ± 9.569	2.677 ± 0.2631	0.9698 ± 0.3648
DOX	47.98 ± 7.086 ***	23.71 ± 3.191 ***	3.810 ± 0.5565 **	2.919 ± 0.5509 ***
DOX + CAL	74.44 ± 10.89 **	46.00 ± 10.65 *	2.960 ± 0.3513 *	1.610 ± 0.4335 **
DOX + CAL + ML385	52.06 ± 13.79 ^#^	26.53 ± 8.909 ^#^	3.853 ± 0.3700 ^#^	2.580 ± 0.3789 ^#^

In contrast to the group DOX, * *p* < 0.05, ** *p* < 0.01, *** *p* < 0.001. ^#^ *p* < 0.05 vs. DOX + CAL group.

## Data Availability

The data supporting the conclusions of this study are included within the article. Additional data are available from the corresponding author upon reasonable request.
